# Interplay Between Phenotype and Genotype in Multidrug‐Resistant and Hypervirulent *Klebsiella pneumoniae* Isolates From Southern Bangladesh

**DOI:** 10.1155/cjid/2610958

**Published:** 2026-10-17

**Authors:** Nazia Farha, Md. Jahidul Islam Noman, Muhammed Jamil Zulqiful, Rinvi Nusrat Prapti, Kallyan Chakma, Sumaiya Alam Omi, Farjana Sharmen, Nahid Sultana, Sanjoy Kanti Biswas, M. Jalal Uddin, Afroza Akter Tanni, Adnan Mannan, Md. Mahbub Hasan

**Affiliations:** ^1^ Drug Discovery and Development Laboratory Department of Genetic Engineering and Biotechnology Faculty of Biological Sciences University of Chittagong Chattogram 4331 Bangladesh cu.ac.bd; ^2^ Department of Genetic Engineering and Biotechnology Faculty of Biological Sciences University of Chittagong Chattogram 4331 Bangladesh cu.ac.bd; ^3^ Department of Microbiology Chattogram Maa-O-Shishu Hospital Medical College Chattogram 4100 Bangladesh; ^4^ Department of Community Medicine Chattogram Maa-O-Shishu Hospital Medical College Chattogram 4100 Bangladesh

**Keywords:** antimicrobial resistance (AMR), Bangladesh, hypervirulent strain, *Klebsiella pneumoniae*, multidrug resistance (MDR)

## Abstract

*Klebsiella pneumoniae* (Kpn) is an opportunistic bacterium associated with various diseases, with significant morbidity and mortality due to treatment challenges posed by hypervirulent and multidrug‐resistant (MDR) strains. This study investigates the prevalence of antimicrobial resistance, virulence factors, and clonal diversity of Kpn isolates. A total of 704 records were collected from the patient registries of two tertiary‐care hospitals in Chattogram, Bangladesh. From these records, 123 biochemically confirmed clinical *Klebsiella species* were selected from admitted patients for molecular analysis. Antibiotic susceptibility, hypermucoid phenotype, and the presence of AMR genes (*sul1*, *aadB*) and the hypervirulence gene (*rmpA*) were determined. Whole‐genome sequencing was also performed on one of the most drug‐resistant isolates. Out of all isolates (*n* = 704), 72.59% were MDR, 18.89% DDR, and 7.95% DSDR. Among MDR, hospital‐acquired infections (76%) were more prevalent than community‐acquired infections (24%), with higher rates among geriatric (27%) and pediatric (25%) patients. The isolates showed high resistance to third‐generation cephalosporins and the highest sensitivity to colistin. PCR analysis of the 81 *phoE-*positive isolates revealed that 85.18% carried the *sul1* gene, while 6.17% harbored the *aadB* gene. Additionally, 12.34% were positive for the virulence gene *rmpA*, and 1.24% contained all three genes, *sul1, aadB,* and *rmpA*. Among the isolates, 65.43% were string test‐positive. Notably, these string test‐positive strains were resistant to most tested antibiotics compared to string test‐negative strains. Whole‐genome sequencing confirmed that KP‐CU‐1 as *K. pneumoniae*, with a 5.26 Mbp draft genome and an N50 of 164,530 bp, classified as sequence type 29 (ST29). This study indicates that MDR Kpn is highly prevalent, with a high proportion of hypervirulent strains. Establishing a nationwide AMR surveillance network will be essential to control the spread of MDR and hypervirulent Kpn in Bangladesh.

## 1. Introduction

Among the most clinically significant multidrug‐resistant (MDR) organisms, *Klebsiella pneumoniae* (Kpn), a Gram‐negative, nonmotile, encapsulated bacterium, is commonly implicated in healthcare‐associated infections. Numerous illnesses, including pneumonia, liver abscess, bacteremia, urinary tract infections, meningitis, and soft tissue infections, are caused by Kpn, especially in young children, the elderly, and people with weakened immune systems [1, 2]. Additionally, it frequently colonizes the gastrointestinal tract and can persist in environmental niches like soil and water bodies, as well as on medical devices [3].

Kpn is divided into two types: hypervirulent *K. pneumoniae* (hvKp) and classical *K. pneumoniae* (cKp). In contrast to cKp, which is mostly linked to infections in healthcare settings, HvKp can cause serious infectious diseases in both healthy people in the community and patients in healthcare settings. Clinically, accurately identifying HvKp is critical becasue it has crucial treatment implications, including helping to locate concealed abscesses for source control and monitoring for endophthalmitis [4]. However, current classical clinical microbiology methods cannot differentiate between these two pathotypes [5]. Unlike cKp, which causes opportunistic nosocomial infections, hvKp strains are more virulent and can disseminate from the primary infection site to distant organs, causing metastatic infections like endophthalmitis, meningitis, necrotizing fasciitis, and osteomyelitis [4]. HvKp is distinguished by the hypermucoviscous phenotype, often confirmed by the string test (viscous string > 5 mm long), and is commonly associated with specific virulence genes such as *rmpA*, *rmpA2*, *magA*, *iucA*, *peg-344*, and *iroB* and siderophores like aerobactin (*iutA*), salmochelin (*iroN*), and yersiniabactin, which enhance iron acquisition and immune evasion. These genes are often located on large virulence plasmids such as pLVKP or pK2044 [6]. While hvKp strains were initially considered to be antibiotic susceptible, increasing evidence indicates a worrying trend of convergence between hypervirulence and multidrug resistance, resulting in highly virulent, drug‐resistant “superbugs” [7, 8]. This convergence presents an unprecedented treatment challenge as strains simultaneously exhibit enhanced virulence and resistance to multiple antibiotic classes, including carbapenems, aminoglycosides, fluoroquinolones, and even colistin.

The acquisition of mobile genetic elements encoding *dfr* and *sul* genes within integrons has also driven resistance to trimethoprim–sulfamethoxazole (TMP–SMX), an inexpensive and widely used antibiotic in environments with limited resources [9]. Aminoglycoside‐modifying enzymes like aminoglycoside acetyltransferase (AAC), aminoglycoside adenylyltransferase (AAD), and aminoglycoside phosphotransferase (APH), which are encoded by genes like *aacC1*, *aadB*, and *aphA6*, can be modified to promote aminoglycoside resistance [10]. A study conducted in Dhaka, Bangladesh, reported that Kpn showed up to 90% resistance to carbapenem antibiotics, including imipenem, with more than 80% of the isolates demonstrating multidrug resistance. The researchers also identified sequence type 15 (ST15) strains that were resistant to colistin, signaling the emergence of pan–drug‐resistant (PDR) organisms [11]. Moreover, higher death rates among pediatric patients with pneumonia and bacteremia have been associated with growing resistance of Kpn [12].

Whole‐genome sequencing (WGS) has become an essential tool for characterizing Kpn sequence types (STs), capsular (K) and lipopolysaccharide (O) loci, antimicrobial resistance determinants, and virulence‐associated genes, enabling the identification of globally disseminated high‐risk clones such as ST11, ST14, ST15, ST147, ST231, and ST307 [12, 13]. WGS has also revealed the convergence of antimicrobial resistance and virulence traits, resulting in highly pathogenic strains with limited treatment options. In Bangladesh, genomic data remain limited although recent studies have identified internationally recognized high‐risk and hypervirulent clones, with bla_CTX-M-15_ as the predominant ESBL gene and O1 as a common serotype [12–14]. Whole‐genome analyses from Dhaka and Chattogram further demonstrate substantial genetic diversity, underscoring the need for continuous genomic surveillance.

The majority of Kpn research in Bangladesh is restricted to phenotypic or molecular characterizations, despite worldwide advances in genomic surveillance. Comprehensive, genome‐based, long‐term research is desperately needed to better understand the virulence characteristics, resistance mechanisms, and clonal diversity of circulating strains. Notably, while the hypermucoviscous string test is widely used as a phenotypic indicator of hvKp, few studies in Bangladesh have investigated the association between string test positivity and antimicrobial resistance. Hence, this study aims to investigate the phenotypic and genotypic interplay between hypervirulent and MDR Kpn isolates from tertiary care hospitals in Chattogram, Bangladesh. This was achieved by integrating epidemiological record analysis, antimicrobial susceptibility profiling, string test, targeted PCR detection of resistance and virulence genes, and WGS of a representative MDR isolate. The findings address a crucial gap in national AMR research and contribute valuable insights to inform clinical management and infection control strategies.

## 2. Methods

### 2.1. Study Design

This study was conducted at two tertiary care hospitals in Chattogram: Chattogram Maa‐O‐Shishu Hospital and Chevron Clinical Laboratory (Pte) Ltd. From the microbiology registry of the respective hospital, all laboratory‐confirmed Kpn cases recorded between November 2023 and August 2024 were included for epidemiological analysis (*n* = 704). Demographic and clinical information associated with these cases was extracted from hospital records to assess risk factors of multidrug resistance. During the same period, clinical specimens from both inpatients and outpatients routinely submitted to the diagnostic microbiology laboratory were prospectively sampled twice weekly. Samples yielding confirmed *Klebsiella* spp. isolates were included in the molecular analysis, resulting in 123 nonduplicate clinical isolates obtained from urine, blood, tracheal aspirate, sputum, pus, wound swabs, and high vaginal swabs. Isolates that could not be confirmed as Kpn or yielded insufficient material for further laboratory analysis were excluded. This study was approved by the Institutional Review Board (IRB) of Chattogram Maa‐O‐Shishu Hospital Medical College (CMOSHMC/IRB/2022/04).

### 2.2. Isolation and Identification of Kpn

Several clinical samples (blood, urine, tracheal aspirate, high vaginal swab, wound swab, sputum, and pus) obtained from patients were cultured and stored at −20°C. MacConkey agar was used to revive bacterial cultures from stock for further analysis. To identify the specific microorganisms, the study used biochemical tests, including Simmon’s Citrate and Triple Sugar Iron (TSI), to detect Kpn.

### 2.3. String Test

The string test can assist in the early diagnosis of hvKp infection. The test is deemed positive when a viscous string longer than 5 mm is produced by stretching bacterial colonies on an agar plate using an inoculation loop or needle [15].

### 2.4. Antimicrobial Susceptibility Testing

The antimicrobial susceptibility pattern of isolated Kpn to several antibiotic classes was examined in vitro using the Kirby–Bauer disc diffusion method. According to the Clinical and Laboratory Standards Institute (CLSI) guideline, susceptibility was determined by measuring the zone of inhibition [16]. To prepare the McFarland standard of the bacterial suspension, the bacteria were cultured on selective media (15 mL of MacConkey, HiMedia) and incubated for 18 h. Following that, pure colonies (2‐3 colonies) were picked and suspended in 1‐mL normal saline adjusted to 0.5 McFarland turbidity standard (0.5 McFarland = 1^∗^108–2^∗^108 CFU/mL) in a 1.5‐mL Eppendorf tube. To prepare the Mueller–Hinton Agar (MHA) plate, 0.113 M of MHA media (38 g/1000 mL, HiMedia) was prepared and poured into Petri dishes, as well as allowed the plate to come to room temperature. Upon solidification of the plates, a sterile swab stick was dipped into the bacterial suspension tube and rotated against the side of the tube to remove the excess fluid. The swab stick was inoculated on the dried surface of the MHA plate by streaking the swab 3 times over the entire agar surface, ensuring the even distribution of inoculum on the plate. Subsequently, the plate was allowed to sit at room temperature for at least 3–5 min, and selective antibiotic discs were positioned on agar plates using sterile forceps. Furthermore, a small and round filter sheet was employed as a negative control. Then, the plate was incubated for 16–18 h at 35°C (35°C ± 2°C) in an air incubator. Following incubation, the zone sizes were measured using a ruler. According to the CLSI guidelines, isolates are classified as susceptible, intermediate, or resistant based on these zone diameters, which are interpreted using established breakpoint tables specific to each antibiotic and organism. Major classes of antibiotics used for the test were cephalosporin, aminoglycoside, fluoroquinolone, carbapenem, tetracycline, polymyxin, penicillin, nitrofuran, macrolide, TMP, SMX, amoxicillin, clavulanic acid, tazobactam, piperacillin, ceftazidime, avibactam, cefoperazone, sulbactam, and ertapenem.

### 2.5. Polymerase Chain Reaction (PCR) Analysis of Antimicrobial Resistance and Virulence Genes

Bacterial genomic DNA was extracted using the heat‐shock method, also known as the boiling method [17]. The presence of the *phoE* gene, a Kpn‐specific chromosomal marker, was first assessed by PCR to molecularly confirm the identity of the isolates. Subsequently, *phoE*‐positive isolates were screened for the antimicrobial resistance genes (*sul1* and *aadB*) and the virulence gene (*rmpA*) using gene‐specific primers (Table S1). Plasmid DNA extraction was conducted utilizing a commercial plasmid DNA extraction kit named FavorPrep Plasmid Extraction Mini Kit, following the manufacturer’s instructions. All PCR steps were done under aseptic conditions in a sterile clean hood using 0.2‐mL PCR tubes (Rnase/DNAse free tubes). Each PCR reaction was prepared in a final volume of 15 μL, consisting of 7.5 μL of PCR master mix, 1 μL each of forward and reverse primers (Table S1), 4.5 μL of nuclease‐free water, and 1 μL of template DNA. Target genes were amplified using specific primers (Table S1), and PCRs were carried out using the optimized PCR profiles (Table S2). Amplified products of PCR were detected by ultraviolet transillumination after electrophoresis on 1.5% agarose gel prepared with 1X TAE buffer and stained with ethidium bromide.

### 2.6. WGS and Genomic Characterization

The selected MDR sample KP‐CU‐1 was subjected to WGS, followed by genomic DNA extraction using the QIAamp DNA extraction kit (QIAGEN, Germany) [18]. The sample’s paired‐end library was prepared using a NEBNext Ultra II FS DNA library prep kit (Illumina, San Diego, CA) according to the manufacturer’s instructions. The library size, quality, and concentration were checked using AMPure XP beads (Beckman Coulter) [19], agarose gel electrophoresis, and a Qubit fluorometer (Thermo Fisher Scientific, USA), respectively [20]. Finally, sequencing was conducted on the Illumina iSeq100 platform using a pair‐end 300‐cycle protocol [13].

Using Trimmomatic v0.40, low‐quality sequence reads and adapter sequences were excluded, and the paired‐end reads were assembled using SPAdes 3.14 [21, 22]. The read quality was assessed using FASTAQC v.0.11.9 [23], and the assembled genome statistics were obtained through QUAST v5.2 [24]. The Kpn genome was taxonomically screened using Kraken2 [25, 26] and analyzed the genome as Kpn with associated factors, STs, antimicrobial resistance genes, and virulence and antigenic factors via the Kleborate tool [27]. Moreover, the genome was visualized in CGview/Proksee alongside CARD data [28] and compared with a reference genome, ASM2354605v1, using BLAST [29]. The genome was further analyzed to identify rRNA and tRNA genes, protein‐encoding genes, and associated functions of the genes through the RAST server [30].

### 2.7. Statistical Analysis

Statistical analyses were performed using Microsoft Excel, RStudio Version 4.2.2, and GraphPad Prism Version 8.0.2. The dataset was summarized using descriptive statistics. To evaluate associations among antibiotic classes, phenotypic characteristics, resistance genes, and demographic variables, Spearman’s rank correlation analysis was performed. Using the corrplot package in *R*, correlation matrices and heatmaps were generated [31]. Statistical significance was determined using the chi‐square test and Fisher’s exact test, with a *p* value < 0.05 considered significant.

## 3. Results

### 3.1. Demographic Distribution and Clinical Characteristics of Kpn Isolates

In this study, we have analyzed hospital records of 704 patients infected with Kpn at Chattogram Maa‐O‐Shishu Hospital Medical College and Chevron Clinical Laboratory (PTE) Ltd. Patient information, including gender, age, sample type, geographic location, and hospital ward of admission, was systematically analyzed. Approximately 24% of the community‐acquired MDR Kpn infections were identified among outpatients, whereas 76% were detected among hospitalized patients across various wards (*p* = 0.034). The prevalence of multidrug resistance was significantly higher among geriatric (≥ 60 years) patients (27%) compared to other age groups (*p* = <0.001). Among all patients infected with Kpn, 21% were children (< 15 years). However, 19%, 18%, and 15% of the patients were classified as young individuals (15–29 years), adults (30–44 years), and older adults (45–59 years), respectively. Additionally, the frequency of MDR Kpn infections was higher in urban areas (58%) than in rural areas (42%) (*p* < 0.001) (Table 1).

**TABLE 1:** Demographic characteristics of patients with *Klebsiella pneumoniae* infections stratified by antimicrobial resistance profile (*N* = 704).

Parameter	MDR^∗^ 72.59% (*N* = 511)	DDR^∗^ 18.89% (*N* = 133)	SDR^∗^ 7.95% (*N* = 56)	Sensitive 0.57% (*N* = 4)	Overall 100% (*N* = 704)	*p* value^∗∗^
*Age (in years)*						
< 15	25% (127)	16% (21)	3.6% (2)	25% (1)	21% (151)	**< 0.001**
15‐ < 30	18% (94)	20% (26)	21% (12)	25% (1)	19% (133)	
30‐ < 45	16% (80)	20% (27)	29% (16)	25% (1)	18% (124)	
45‐ < 60	14% (70)	20% (27)	14% (8)	0% (0)	15% (105)	
> 60	27% (140)	24% (32)	32% (18)	25% (1)	27% (191)	

*Patient care type*						
Inpatients	76% (388)	73% (97)	64% (36)	25% (1)	74% (522)	**0.034**
Outpatients	24% (123)	27% (36)	36% (20)	75% (3)	26% (182)	

*Residence type*						
Rural	42% (140)	22% (18)	27% (8)	—	37% (166)	**< 0.001**
Urban	58% (191)	78% (65)	73% (22)	—	63% (278)	

*Note:* Significant *p* values are presented in bold.

^∗^MDR = multidrug resistant, DDR = double drug resistant, SDR = single drug resistant.

^∗∗^ *p* values were compared among single‐, double‐, and multidrug‐resistant groups.

In hospital‐acquired infections (inpatients), the highest proportion of isolates was obtained from tracheal aspirates (30.46%), followed by sputum (24.9%), urine (19.93%), pus (12.45%), blood (6.13%), wound swabs (4.6%), and high vaginal swabs (1.53%) (Figure 1a). In community‐acquired infections (outpatients), Kpn isolates were most frequently recovered from urine (45.6%), followed by sputum (18.13%), pus (12.08%), wound swabs (7.69%), tracheal aspirates (2.19%), and blood (1.09%), whereas no isolates were obtained from high vaginal swabs (Figure 1b).

**FIGURE 1:**
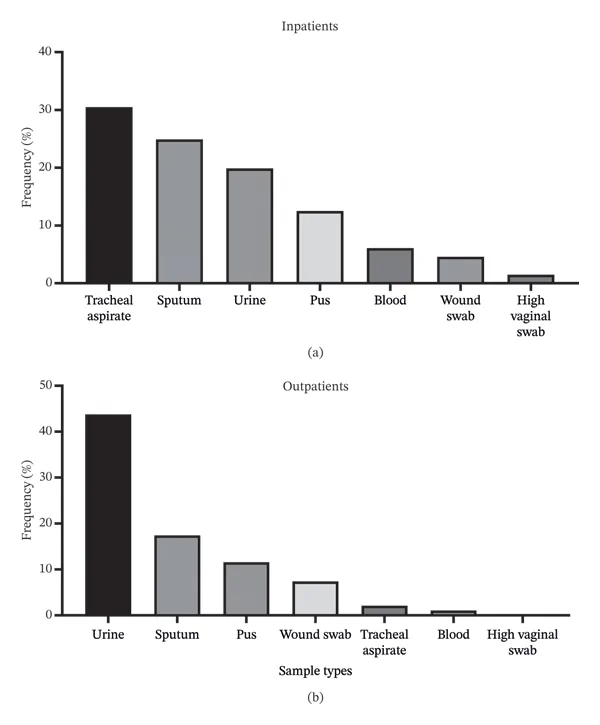
Distribution of *Klebsiella pneumoniae* (Kpn) isolates according to the specimen type among (a) hospitalized patients and (b) outpatients (*N* = 704). Values represent percentage frequencies of isolates recovered from each sample type.

Clinical outcomes of Kpn infections and patients’ admission history were recorded and analyzed, considering variables such as the sample type, hospital ward, and length of hospital stay. MDR isolates were mostly found in tracheal aspirate (30%) and urine (29%) samples. Sputum specimens predominantly harbored both double drug‐resistant (38%) and single drug‐resistant (45%) Kpn strains. In addition, patients who had the most severe Kpn infections were admitted to different wards, including ICU (30%), Medicine (22%), Gynecology (8.8%), Neonatal Intensive Care Unit (5.4%), High Dependency Unit (5.4%), and Pediatric (12%) wards. Moreover, Kpn infections were found in surgery (6.3%), Critical Care Unit (0.4%), Orthopedics (5.5%), Pediatric Intensive Care Unit (0.6%), and Special Care Baby Unit (2.1%), among others (Table 2).

**TABLE 2:** Clinical characteristics of the study population.

Parameter	MDR %: 72.59% (*N* = 511)	DDR %: 18.89% (*N* = 133)	SDR %: 7.95% (*N* = 56)	Sensitive %: 0.57% (*N* = 4)	Overall: 100% (*N* = 704)	*p* value^∗^
*Hospital wards*						
CCU	0.3% (1)	1% (1)	0% (0)	0% (0)	0.4% (2)	**0.034**
ENT	0.3% (1)	0% (0)	0% (0)	0% (0)	0.2% (1)	
Gyne	8.2% (32)	12% (12)	5.6% (0)	0% (0)	8.8% (44)	
HDU	3.6% (14)	0% (0)	2.8% (0)	0% (0)	2.9% (14)	
ICU	35% (138)	11% (11)	19% (7)	0% (0)	30% (156)	
KMC	1% (4)	0% (0)	0% (0)	0% (0)	0.8% (4)	
Medicine	16% (63)	37% (36)	47% (17)	0% (0)	22% (116)	
NICU	6.9% (27)	1% (1)	0% (0)	0% (0)	5.4% (28)	
Oncology and Hematology	1.3% (5)	3.1% (3)	5.6% (2)	0% (0)	1.9% (10)	
Orthopedic	5.1% (20)	7.2% (7)	2.8% (1)	0% (0)	5.5% (28)	
Pediatrics	13% (51)	12% (12)	2.8% (1)	0% (0)	12% (64)	
PICU	0.8% (3)	0% (0)	0% (0)	0% (0)	0.6% (3)	
SCBU	2.3% (9)	2.1% (2)	0% (0)	0% (0)	2.1% (11)	
Surgery	4.6% (18)	11% (11)	11% (4)	0% (0)	6.3% (33)	
Urology	0.8% (3)	1% (1)	2.8% (1)	0% (0)	1% (5)	

*Sample type*						
Blood	5.6% (28)	4.7% (6)	0% (0)	0% (0)	5% (34)	—
High vaginal swab	0.8% (4)	1.6% (2)	4.1% (2)	0% (0)	1.2% (8)	
Pus	11% (57)	16% (20)	18% (9)	100% (1)	13% (87)	
Sputum	18% (92)	38% (49)	45% (22)	0% (0)	24% (163)	
Tracheal aspirate	30% (151)	7% (9)	6.1% (3)	0% (0)	24% (163)	
Urine	29% (144)	25% (32)	22% (11)	0% (0)	28% (187)	
Wound swab	5% (25)	8.5% (11)	4.1% (2)	0% (0)	5.6% (38)	

*Note:* MDR = multidrug resistant. Significant *p* values are presented in bold.

Abbreviations: DDR = double drug resistant, SCBU = special care baby unit, SDR = single drug resistant.

^∗^ *p* values were compared among single‐, double‐, and multidrug‐resistant groups.

### 3.2. MDR Patterns of Kpn Isolates

A concerning percentage of Kpn strains exhibited resistance to several antibiotics, rendering them ineffective for treatment. Ceftazidime, cefuroxime, and cefixime had the highest resistance. More than 80% of the bacteria tested positive for resistance to them. Colistin and nitrofurantoin exhibited sensitivity rates of > 70%, whereas the remaining antibiotics (ceftriaxone, cefepime, cefuroxime, cefixime, and ceftazidime) from various classes showed sensitivity rates of < 30%. The highest resistance was observed in adults, with rates of 40.19% for cefuroxime, 33.94% for cefixime, 30.68% for ceftriaxone, and 29.98% for cefepime. In children, resistance was recorded as 19.46% (cefuroxime), 14.77% (cefixime), 17.18% (ceftriaxone), 15.48% (cefepime), 12.64% (ciprofloxacin), 9.80% (gentamicin), 8.66% (amikacin), 9.51% (levofloxacin), and 9.09% (cotrimoxazole). Among geriatric patients, resistance was 21.73% (cefuroxime), 18.89% (cefixime), 19.17% (ceftriaxone), 18.60% (cefepime), 14.06% (ciprofloxacin), 9.65% (gentamicin), 10.36% (amikacin), 11.50% (levofloxacin), and 8.80% (cotrimoxazole). Resistance to both cefuroxime and cefepime was highest in adults, reaching 40.19% and 29.98%, respectively, compared with 21.73% and 18.60% in geriatric patients and 19.46% and 15.48% in children (Figure 2).

**FIGURE 2:**
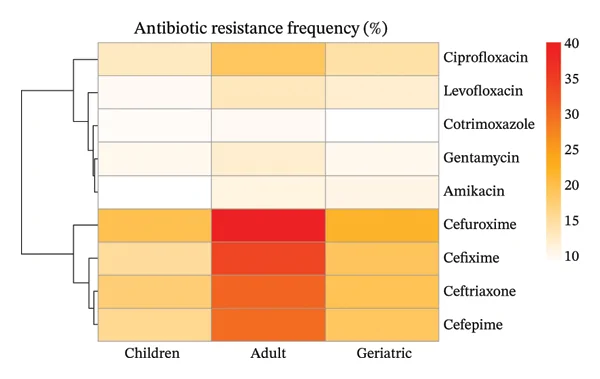
Heatmap showing the percentage resistance of *K. pneumoniae* isolates to selected antibiotics across children, adults, and geriatric age groups.

To confirm pure Kpn isolation from clinical samples, the housekeeping gene *phoE* (outer membrane phosphoporin E) was targeted using conventional PCR. From 123 culture‐confirmed *Klebsiella* isolates, we found a total of 81 *phoE*‐positive isolates. These *phoE-*positive Kpn isolates were selected for investigating the molecular basis of Kpn infections (Figure 3 and Table 3).

**FIGURE 3:**
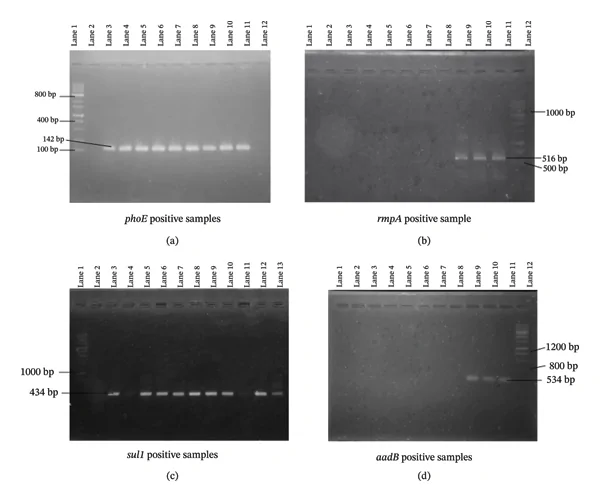
PCR‐based detection of different *K. pneumoniae* genes: (a) housekeeping gene *phoE* (Lane 1: Ladder, Lane 3–11: *phoE* positive), (b) hypervirulence‐associated *rmpA* (Lane 12: Ladder, Lane 9–11: *rmpA* positive), (c) MDR‐associated *sul1* (Lane 1: Ladder, Lane 3, 5–10, 12–13: *sul1* positive), and (d) *aadB* (Lane 12: Ladder, Lane 9–11: *aadB* positive).

**TABLE 3:** Distribution of antimicrobial resistance (*sul1*, *aadB*) and virulence (*rmpA*) genes among *phoE*‐positive *Klebsiella pneumoniae* isolates (*n* = 81).

Parameter	*sul1*	*aadB*	*rmpA*	Overall MDR (*sul1, aadB*)	MDR (*sul1, aadB*) and hypervirulent gene (*rmpA*)
	85.18%; *N* = 69	6.17%; *N* = 5	12.34%; *N* = 10	4.94%; *N* = 4	1.24%; *N* = 1
*Age group (years)*					
< 15	9.88% (8)	0% (0)	2.47% (2)	0% (0)	0% (0)
15‐ < 30	7.41% (6)	0% (0)	0% (0)	0% (0)	0% (0)
30‐ < 45	9.88% (8)	0% (0)	2.47% (2)	0% (0)	0% (0)
45‐ < 60	27.16% (22)	3.70% (3)	2.47% (2)	3.70% (3)	0% (0)
> 60	30.86% (25)	2.47% (2)	4.94% (4)	1.24% (1)	1.24% (1)

*Patient type*					
Inpatients	67.90% (55)	4.94% (4)	12.34% (10)	3.70% (3)	1.24% (1)
Outpatients	17.28% (14)	1.24% (1)	0% (0)	1.24% (1)	0% (0)

*Sample type*					
Urine	17.28% (14)	2.47% (2)	2.47% (2)	2.47% (2)	1.24% (1)
Tracheal aspirate	53.09% (43)	1.24% (1)	8.64% (7)	1.24% (1)	0% (0)
Sputum	6.17% (5)	1.24% (1)	1.24% (1)	0% (0)	0% (0)
Blood	3.70% (3)	0% (0)	0% (0)	0% (0)	0% (0)
Pus	2.47% (2)	1.24% (1)	0% (0)	1.24% (1)	0% (0)
Wound swab	2.47% (2)	0% (0)	0% (0)	0% (0)	0% (0)

*Note:* Percentages are calculated based on the total number of *phoE*‐positive isolates (*n* = 81). Because some isolates carried multiple genes, the frequencies of individual genes are not mutually exclusive.

Antimicrobial resistance–associated gene *sul1* was present in 85.18% of PCR‐confirmed Kpn isolates. The *sul1* gene‐positive isolates were primarily found in hospitalized patients (67.9%) and in the age group > 60 years (30.86%). Tracheal aspirate (53.09%) was the most frequent type of sample to have Kpn with the *sul1* gene (Figure 3C and Table 3). Similarly, the *aadB* gene was screened and found in only 6.17% of Kpn isolates. The gene was detected at a higher rate in hospitalized patients (4.94%) and was found among individuals aged 45‐< 60 years (3.70%). Urine samples were the most frequent source of Kpn isolates carrying the *aadB* gene (2.47%) (Figure 3D and Table 3). Moreover, the hypervirulent *rmpA* gene was detected in only 12.34% of Kpn isolates. The gene was predominantly identified in hospitalized individuals (12.34%) and was most associated with patients over 60 years of age (4.94%). Among clinical samples, tracheal aspirates exhibited the highest proportion of Kpn isolates carrying the *rmpA* gene (8.64%) (Figure 3 and Table 3).

### 3.3. String Test to Identify the Hypermucoid Phenotype

A sterile platinum wire loop was used in a string test to lift a colony of Kpn cultured on a culture plate to identify the hypermucoid phenotype. A total of 65.43% (*N* = 53) of the string test‐positive samples were found to be *phoE*‐positive Kpn isolates. Among string test‐positive isolates, 81.13% carried *sul1*, 7.55% carried *aadB*, and 7.41% carried the hypervirulence gene *rmpA*. Most of the string test‐positive samples (41.97%) were isolated from tracheal aspirate (Figure S1 and Table S3).

### 3.4. Spearman’s Correlation Matrix Analysis

Several noteworthy correlations between antibiotic classes, phenotypic features, resistance determinants, and demographic factors were found by correlation analysis (Figure 4). Consistent with their established functions, inducing antimicrobial resistance, resistance genes *sul1* and *aadB* demonstrated positive associations with resistance to aminoglycosides and fluoroquinolones. Across several antibiotic classes, string test positivity and the hypermucoviscosity‐associated marker *rmpA* showed somewhat positive associations with resistance across multiple antibiotic classes.

**FIGURE 4:**
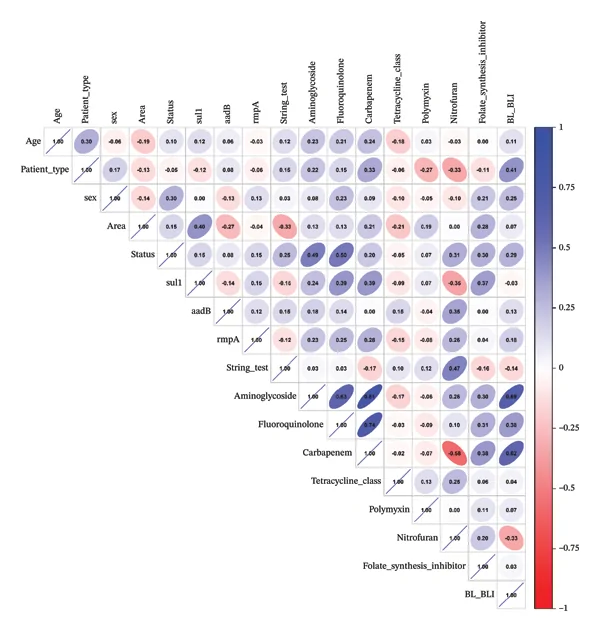
Spearman’s correlation plot showing associations among demographic variables, resistance genes, phenotypic traits, and antibiotic classes. Spearman’s correlation coefficients (*r*) are displayed within each circle, with circle size and color intensity proportional to the strength of the correlation. Blue circles represent positive correlations, whereas red circles represent negative correlations. The color scale ranges from −1 (strong negative correlation, red) to +1 (strong positive correlation, blue). BL_BLI indicates β‐lactam/β‐lactamase inhibitor.

There were strong positive associations between related antibiotic classes, especially between fluoroquinolones and aminoglycosides, suggesting possible coresistance tendencies. This finding may indicate overlapping resistance mechanisms or common selection pressure; carbapenem resistance exhibited positive correlations with β‐lactam/β‐lactamase inhibitor (BL_BLI) combinations. On the other hand, nitrofuran resistance showed a clear pattern of resistance, with weak or negative correlations with the majority of other antibiotic classes. Age, sex, patient type, and place of residency were among the demographic variables that exhibited weak to moderate associations with resistance features. This suggests that host factors have a limited but discernible impact on the spread of antimicrobial resistance.

### 3.5. Whole‐Genome Analysis of MDR Kpn

#### 3.5.1. Gene Annotation and Subsystem Categorization

The whole genome of isolate KP‐CU‐1 was further confirmed as Kpn, resulting in a draft assembly size of 5.26 Mbp with an N50 of 164,530 bp. Multilocus sequence typing (MLST) classified this isolate as Sequence Type 29 (ST29), a lineage commonly linked to clinical infections. *In silico* serotyping with Kleborate identified the capsular locus as KL54 and the lipopolysaccharide locus as O1.

The isolate is characterized as a MDR strain, with a Kleborate resistance score of 1, indicating the presence of extended‐spectrum *β*‐lactamases (ESBLs). Its resistome includes the globally widespread ESBL gene bla*CTX-M-15, alongside bla*TEM‐1D and the chromosomal bla_SHV‐187. Beyond *β*‐lactam resistance, the isolate carries acquired resistance genes that confer reduced susceptibility to several other antimicrobial classes, including fluoroquinolones (qnrS1), aminoglycosides (strA and strB), sulfonamides (sul2), and tetracyclines (tet(A)). No carbapenemase genes were detected. Although phenotypic characterization showed a hypermucoviscous profile with a positive string test, genomic analysis using Kleborate assigned a Virulence Score of 1. This score reflects the presence of yersiniabactin (ybt 14 on ICEKp5) but the absence of key hypervirulence‐associated loci such as aerobactin (iuc), salmochelin (iro), and rmpA/A2 regulators. Therefore, KP‐CU‐1 is classified as an ESBL‐producing, hypermucoviscous cKp, underscoring the emergence of enhanced phenotypic virulence within a MDR clinical lineage.

The whole genome of KP‐CU‐1 was annotated and categorized into 573 subsystems via the RAST server. Most of the annotated genes found were functioning in carbohydrates (421), amino acids and derivatives (401), cofactors, vitamins, prosthetic groups and pigments (192), protein metabolisms (217), RNA metabolisms (53), membrane transport (97), cell wall and capsule (36), and iron acquisition and metabolism (39), which are crucial for bacterial survival and adaptation. In addition, the genomes carry 55 genes responsible for virulence, disease, and defense, causing lethal effects in humans (Figure 5a).

**FIGURE 5:**
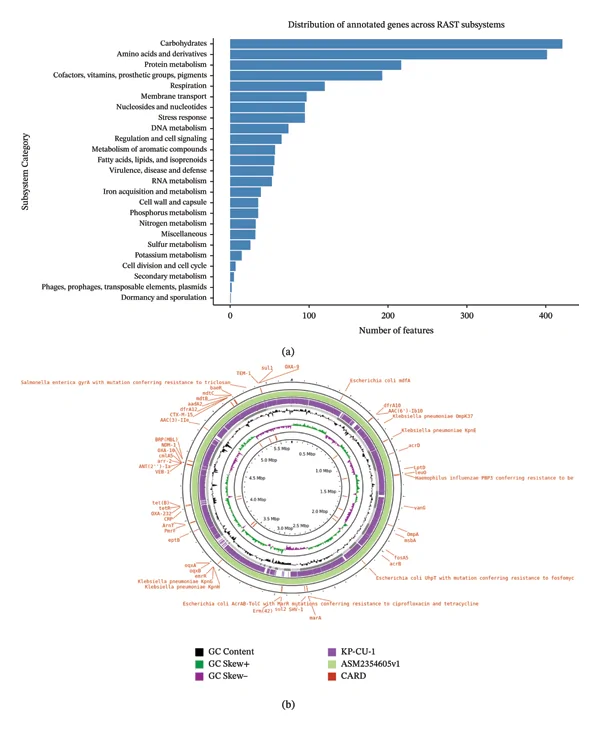
Genomic landscape and subsystem distribution of *Klebsiella pneumoniae* KP‐CU‐1. (a) Distribution of RAST subsystem categories showing the feature counts of annotated genes across the KP‐CU‐1 genome. (b) Schematic circular representation of the 5.26‐Mbp KP‐CU‐1 genome. From the innermost to the outermost track: Mbp scale; Comprehensive Antibiotic Resistance Database (CARD) annotations (red); GC skew (positive in green, negative in purple); GC content (black); gene locations for KP‐CU‐1 (violet) and reference ASM2354605v1 (light green); and specific resistance genes (e.g., *sul1*, *gyrA*, OXA‐9, and tetR) identified across multiple genera (*Klebsiella*, *Escherichia*, *Salmonella*, and *Haemophilus*) mapped to their respective genomic positions.

#### 3.5.2. Comparative Genomic Analysis of MDR Kpn Strain KP‐CU‐1

The analysis of the Kpn genome KP‐CU‐1, along with related bacterial species, reveals a diverse array of antibiotic resistance genes, annotated in red with genomic position markers. Key findings include triclosan resistance from a *Salmonella enterica gyrA* mutation, multidrug resistance via *Escherichia coli mdfA*, and efflux pump‐related genes (*ndvB*, *aaeR*, *mdtC*, and *mdtB*). Resistance to TMP and beta‐lactams is noted with *dfrA12*, CTX‐M‐15, and AAC(6′)‐Ib10, while Kpn *ompK37* and KpnE contribute to outer membrane and efflux resistance. Additional genes like BRP (MBL), OXA‐9, *tetR*, ANT(3″)‐Ia, VEB‐1, *sul1*, erm(42), and *marA* confer resistance to beta‐lactams, tetracycline, aminoglycosides, sulfonamides, and macrolides. Furthermore, *E. coli* AcrAB‐TolC with *marR* mutations and *Haemophilus influenzae* PBP3 provide ciprofloxacin, tetracycline, and beta‐lactam resistance, while *vanG*, *fosA5*, *acrB*, and *E. coli uhpT* mutations enable resistance to vancomycin, fosfomycin, and additional multidrug profiles. This extensive resistance profile underscores the adaptability and clinical challenge posed by these pathogens (Figure 5b).

## 4. Discussion

In this study, 72.59%, 18.89%, and 7.95% of Kpn isolated from different clinical specimens were found to be MDR, SDR, and DDR, respectively (Table 1). A similar finding was observed by Aminul et al. among 150 Kpn, 123 (82%) were identified as MDR, and 27 (18%) were identified as non‐MDR [32]. Furthermore, our earlier study in Bangladesh reported that 68.4% of Kpn isolates obtained from various clinical samples were MDR, where 64.73% were found in community‐acquired MDR Kpn*-*infected patients and 71.48% were found in hospital‐acquired infected patients [33]. In this study, we also found that hospital‐acquired infection (76%) is more than community‐acquired cases (24%). Kawser et al. reported that among Kpn isolates recovered from various clinical specimens at the Dhaka Medical College Hospital, Bangladesh, 34.09% were MDR, 22.73% were extensively drug resistant (XDR), and 2.27% were PDR [6].

The highest prevalence was among geriatric patients (27%), followed by children (21%), young adults (19%), adults (18%), and older adults (15%). Urban populations carried a higher burden (58%) compared to rural areas (42%) (Table 1). MDR isolates were mostly recovered from tracheal aspirates (30%) and urine (29%), with hospital‐acquired infections dominated by tracheal aspirates (30.5%) and sputum (24.9%). Severe infections were frequently associated with ICU (30%) and Medicine wards (22%) (Table 2). Similar observations regarding specimen sources have also been identified in ICU units of the surgery, gynecology, obstetrics, and geriatric wards of hospitals in China [34]. In our previous study conducted in Chattogram, Bangladesh, in 2021, Kpn infections were most frequently detected in urine samples (39.9%) among patients younger than 15 years [33]. This may explain the high incidence of lung infections, cough, breathing difficulties, and urinary tract infections in the majority of patients, originating from the community or acquired in hospital settings. As a result, they are either admitted to hospital for treatment or prescribed various medicines. Due to mismanagement or dissemination of MDR bacterial strains in hospital settings, the treatment cost for patients infected with MDR Kpn strains has been a global burden [35, 36].

We determined the highest resistance against ceftazidime (95.3%), cefuroxime (89.3%), cefixime (82.6%), cefotaxime (79.9%), cefepime (78.2%), and ceftriaxone (70.5%). Moderate resistance was observed against imipenem (48.5%), ciprofloxacin (47.2%), azithromycin (45.5%), and cotrimoxazole (43.9%), while the lowest resistance was to colistin (7.2%) (Figure 2). Previous studies in Bangladesh have reported high resistance rates among isolated Kpn strains to commonly used antibiotics such as cefuroxime, ceftriaxone, ceftazidime, cefepime, and ciprofloxacin [37]. Resistance patterns have also been shown to vary across patient age groups, with adults exhibiting the highest resistance rates (up to 40.2% for cefuroxime), compared to children (≤ 19.5%) and geriatric patients (≤ 21.7%) (Figure 2).

In this study, 65.43% of the isolated Kpn were string test positive (Table 3). A study in Dhaka, Bangladesh, found 47.73% string test‐positive Kpn strains [6]. In Egypt, another study reported 40.71% string test‐positive Kpn [38]. The dissimilarity may be due to time, the source of sample collection, and geographical variation. Although hypermucoviscosity is associated with virulence, nonhypermucoviscous or string test‐negative Kpn isolates may also possess the virulence genes and show higher AMR [39]. From this, it can be presumed that there are other factors, such as K1 and K2 capsular serotypes; ST23 and CC23 STs; virulence plasmid pLVPK and KPHP1208 pathogenicity island; or other virulence genes that might be responsible for higher resistance rates [40]. In the present study, virulence gene *rmpA* was detected in 12.34% of the isolated Kpn*-positive* strains (Table 3). Similarly, a recent study from India investigating virulence factors among carbapenem‐resistant and urinary MDR Kpn isolates reported a high prevalence of the virulence genes *fimH* and *entB*, whereas *rmpA* was absent in carbapenem‐resistant isolates and occurred at a low frequency (5.1%) among urinary MDR Kpn strains [41]. Compared with these findings, the present study detected *rmpA* in 12.34% of the PCR‐confirmed Kpn isolates, indicating regional variation in the distribution of hypervirulence‐associated determinants. In a study in Dhaka, virulence gene *rmpA* was detected in 52.38% of the isolated string test‐positive strains. This finding agrees with the study findings in China and Egypt that reported the presence of these genes in 44.4% and 56.52% of the isolated Kpn, respectively [38, 42]. These findings correlate with the literature that *rmpA/rmpA2* activate the *cps* transcription and make the strains more virulent because of capsular polysaccharide synthesis, processing, and movement on the cell surface with the help of the chromosomal *cps* gene cluster in Kpn, especially K1 and K2 capsular serotypes. Moreover, *rmpA* and the derivative gene *rmpA2* are associated with liver abscess caused by the Kpn strains [43].

As shown in Figure 4, resistance genes (*sul1* and *aadB*) were positively associated with resistance to multiple antibiotic classes, supporting their role in the dissemination of multidrug resistance and suggesting possible coselection through mobile genetic elements [44–46]. In addition, string‐test positivity and the virulence‐associated gene *rmpA* showed positive associations with antimicrobial resistance traits, highlighting the growing convergence of resistance and virulence in Kpn [47–49]. The positive associations observed between *rmpA*/string‐test positivity and antimicrobial resistance should be interpreted with caution. As this study is observational and based on correlation analyses, the findings do not establish a causal relationship between virulence‐associated traits and antimicrobial resistance. Strong correlations among several antibiotic classes, particularly fluoroquinolones and aminoglycosides, further suggest shared resistance mechanisms or selective pressures [50, 51]. In contrast, nitrofuran resistance exhibited a distinct correlation pattern, indicating that its underlying resistance mechanisms may differ from those associated with other antimicrobial classes [12, 52]. Collectively, these findings emphasize the complexity of resistance evolution in clinical Kpn populations and reinforce the importance of integrated molecular surveillance.

In healthcare settings, where the use of one antibiotic class may inadvertently select for resistance to another, this phenomenon is often attributed to common selective pressures or overlapping resistance mechanisms [50, 51]. With weak or negative correlations with the majority of other antibiotic classes, nitrofuran resistance demonstrated a distinct pattern [53]. This implies that resistance mechanisms of nitrofuran may be different from those associated with other antibiotic classes, perhaps involving altered expression of membrane transport proteins (*ompK*36), efflux pump (*acr*AB and *oqx*R), the presence of resistance determinants positioning in transferable plasmids, and changes in chromosomal nitroreductase encoding genes (*rib*C and *nfs*B) in Kpn [53]. This observed distinct resistance profile is perhaps a result of the unique mechanism of action of nitrofurans, which requires nitroreduction‐dependent prodrug activation [53, 54].

The whole‐genome analysis of KP‐CU‐1 revealed an ESBL‐producing MDR Kpn belonging to ST29, carrying the globally disseminated *bla*CTX‐M‐15 gene alongside additional determinants conferring resistance to fluoroquinolones, aminoglycosides, sulfonamides, and tetracyclines. The predominance of CTX‐M‐15 is consistent with reports identifying this enzyme as one of the most widespread ESBLs associated with nosocomial and MDR Kpn lineages worldwide [14, 55]. Moreover, the isolate was resistant to ESBL phenotypes, indicated by resistance to ceftazidime, cefuroxime, cefixime, and ceftriaxone. Interestingly, KP‐CU‐1 exhibited a hypermucoviscous phenotype despite lacking the canonical hypervirulence regulators (*rmpA/rmpA2*) and aerobactin loci. Instead, the isolate carried the yersiniabactin (ybt) siderophore cluster located on ICEKp5, a virulence determinant that has been associated with enhanced fitness, iron acquisition, and increased pathogenic potential in clinical Kpn strains [56]. The identification of ST29 is noteworthy because this lineage has previously been reported among both MDR and hypermucoviscous clinical isolates carrying *bla*CTX‐M‐15 and yersiniabactin‐mediated virulence traits. In addition, this strain harbors MDR plasmids that may facilitate the acquisition of more resistance genes or mobile genetic elements into MDR_Hv‐Kpn or carbapenem resistance Hv‐Kpn strains, including K1, K2, and K57 [57]. Notably, WGS identified the isolate as ST29‐KL54/O1 and revealed the presence of *bla*CTX‐M‐15, which is consistent with the observed extended‐spectrum *β*‐lactam resistance phenotype. Previous studies have reported that the ST29‐KL54 lineage is associated with hypervirulence‐related characteristics, including enhanced capsule synthesis, biofilm formation, iron acquisition systems, and hypermucoviscosity [58]. Collectively, these findings suggest that KP‐CU‐1 represents a classical MDR Kpn strain with enhanced virulence‐associated features rather than a true hypervirulent genotype, highlighting the ongoing convergence of resistance and virulence traits that may complicate the clinical management and epidemiological control of Kpn infections.

A strength of this study is the inclusion of a substantial number of epidemiological records (*n* = 704), providing a broad overview of the local burden. However, laboratory analysis was limited to 123 clinical Kpn samples, which may not fully represent the national scenario. Therefore, while the findings provide important insights, they should be interpreted with caution and cannot be generalized at the national level.

## 5. Conclusions

Kpn represents a major public health concern owing to its increasing multidrug resistance, emergence of hypervirulent strains, and association with severe healthcare‐associated infections. We identified a proportion of Kpn isolates carrying hypervirulence‐associated traits, including *rmpA* positivity and hypermucoviscosity, which is of particular concern and needs to be addressed immediately. The presence of difficult‐to‐treat carbapenem‐producing Kpn strains needs more attention to prevent their dissemination. The consistency in population structure (prevalence of high‐risk and hypervirulent lineages) with global isolates puts Bangladesh in the global context in terms of exchange of significant epidemiological Kpn strains, suggesting the potential dissemination of clinically significant Kpn strains across geographical boundaries. These findings emphasize the importance of continuous molecular surveillance, antimicrobial stewardship, and coordinated public health strategies to monitor and limit the spread of MDR and hvKp in Bangladesh.

## Author Contributions

Nazia Farha: laboratory work, data entry, data curation, data analysis, software, visualization, and writing–original draft; Md. Jahidul Islam Noman: data entry, data analysis, software, visualization, and writing–original draft; Muhammed Jamil Zulqiful: laboratory work, data entry, and data curation; Rinvi Nusrat Prapti: data analysis, visualization, and writing–original draft; Kallyan Chakma: software, data analysis, visualization, data curation, and writing–original draft; Sumaiya Alam Omi: data analysis, visualization, and writing–original draft; Farjana Sharmen: conceptualization, supervision, and funding acquisition; Nahid Sultana, Sanjoy Kanti Biswas, and M. Jalal Uddin: conceptualization, project administration, supervision, and validation; Afroza Akter Tanni: laboratory work, data analysis, visualization, resources, data curation, validation, and writing–review and editing; Adnan Mannan: conceptualization, methodology, project administration, resources, funding acquisition, supervision, validation, and writing–review and editing; Md. Mahbub Hasan: conceptualization, methodology, data curation, project administration, supervision, funding acquisition, resources, validation, and writing–review and editing.

## Funding

This study was partially funded by the Ministry of Science and Technology, Government of Bangladesh (Memo No. MOST‐SRG‐221197).

## Disclosure

All authors have reviewed and approved the final manuscript.

## Ethics Statement

This study was approved by the Institutional Review Board (IRB) of Chattogram Maa‐O‐Shishu Hospital Medical College (CMOSHMC/IRB/2022/04). No direct patient contact was involved. Clinical information was obtained from hospital records, and bacterial isolates were collected from the Microbiology Department. All patient data were anonymized prior to analysis to ensure confidentiality.

## Conflicts of Interest

The authors declare no conflicts of interest.

## Supporting Information

Additional supporting information can be found online in the Supporting Information section.

## Supporting information


**Supporting Information** Supporting 1. Figure S1: String test demonstrating hypermucoviscosity in *K. pneumoniae*. Supporting 2. Table S1: Details of primer sequences utilized in this study. Supporting 3. Table S2: PCR amplification conditions for the target genes investigated in this study. Supporting 4. Table S3: Distribution and frequency of string test‐positive *Klebsiella* isolates among different clinical sample types.

## Data Availability

The data that support the findings of this study are available from the corresponding author upon reasonable request.
