# Supplementary material for: Interplay Between Phenotype and Genotype in Multidrug‐Resistant and Hypervirulent Klebsiella pneumoniae Isolates From Southern Bangladesh
Source: Can J Infect Dis Med Microbiol. 2026 Oct 17;2026:2610958. doi: 10.1155/cjid/2610958 (PMC13676301; doi:10.1155/cjid/2610958)
Supplement: Supplementary file 1 [file CJID-2026-2610958-s001.docx]

**Interplay Between Phenotype and Genotype in Multidrug-Resistant and Hypervirulent *Klebsiella pneumoniae* Isolates from Southern Bangladesh**

Nazia Farha^1^, Md. Jahidul Islam Noman^1^, Muhammed Jamil Zulqiful^1^, Rinvi Nusrat Prapti^1^, Sumaiya Alam Omi^1^, Kallyan Chakma^2^, Farjana Sharmen^2^, Nahid Sultana^3^, Sanjoy Kanti Biswas^3^, M. Jalal Uddin^4^, Afroza Akter Tanni^2^, Adnan Mannan^2^ and Md. Mahbub Hasan^1,2,*^

^1^Drug Discovery and Development Laboratory, Department of Genetic Engineering and Biotechnology, Faculty of Biological Sciences, University of Chittagong, Chattogram 4331, Bangladesh

^2^Department of Genetic Engineering and Biotechnology, Faculty of Biological Sciences, University of Chittagong, Chattogram 4331, Bangladesh

^3^Department of Microbiology, Chattogram Maa-O-Shishu Hospital Medical College, Chattogram 4100, Bangladesh

^4^Department of Community Medicine, Chattogram Maa-O-Shishu Hospital Medical College, Chattogram 4100, Bangladesh

*Corresponding author: [mahbub.hasan@cu.ac.bd](mailto:mahbub.hasan@cu.ac.bd)

       

**Figure S1: String test demonstrating hypermucoviscosity in *K. pneumoniae*.** When *K. pneumoniae* colonies were stretched, a string longer than 5 mm was formed, indicating the hypermucoviscous phenotype.

**Table S1: Details of Primer Sequences utilised in this study.** Sources indicate the references from which the PCR primers were adapted.

| **Target Gene** | **Primer sequence (5’-3’)** | **Amplicon size(bp)** | **Location** | **Sources** |
| --- | --- | --- | --- | --- |
| *phoE* F | TGCCCAGACCGATAACTTTA | 142 | Chromosome | [1] |
| *phoE* R | CTGTTTCTTCGCTTCACGG |  |  |  |
| *sul1* F | CTTCGATGAGAGCCGGCGGC | 436 | Plasmid | [2] |
| *sul1* R | GCAAGGCGGAAACCCGCGCC |  |  |  |
| *aadB* F | ATGGACACAACGCAGGTCGC | 534 | Plasmid | [3] |
| *aadB* R | TTAGGCCGCATATCGCGACC |  |  |  |
| *rmpA* F | ACTGGGCTACCTCTGCTTCA | 516 | Plasmid | [4] |
| *rmpA* R | CTTGCATGAGCCATCTTTCA |  |  |  |

**Table S2: PCR amplification conditions for the target genes investigated in this study.** Sources indicate the references from which the PCR conditions were adapted.

| **Target gene** | **Steps** | **Temperature** | **Time** | **Cycle** | **Sources** |
| --- | --- | --- | --- | --- | --- |
| *phoE* | Initial denaturation | 94 | 5 min | 1 | [1] |
|  | Denaturation | 94 | 30 sec | 35 |  |
|  | Annealing | 58.5 | 30 sec |  |  |
|  | Elongation | 72 | 2 min |  |  |
|  | Final elongation | 72 | 5 min | 1 |  |
|  | Hold | 4 | ∞ | 1 |  |
| *sul1* | Initial denaturation | 94 | 5 min | 1 | [2] |
|  | Denaturation | 94 | 30 sec | 35 |  |
|  | Annealing | 58.5 | 30 sec |  |  |
|  | Elongation | 72 | 2 min |  |  |
|  | Final elongation | 72 | 5 min | 1 |  |
|  | Hold | 4 | ∞ | 1 |  |
| *aadB* | Initial denaturation | 95 | 5 min | 1 | [3] |
|  | Denaturation | 94 | 1 min | 30 |  |
|  | Annealing | 58 | 1 min |  |  |
|  | Elongation | 72 | 2 min |  |  |
|  | Final elongation | 72 | 5 min | 1 |  |
|  | Hold | 4 | ∞ | 1 |  |
| *rmpA* | Initial denaturation | 94 | 5 min | 1 | [4] |
|  | Denaturation | 94 | 1 min | 35 |  |
|  | Annealing | 58 | 1 min |  |  |
|  | Elongation | 72 | 1 min |  |  |
|  | Final elongation | 72 | 7 min | 1 |  |
|  | Hold | 4 | ∞ | 1 |  |

**Table S3: Distribution and frequency of string test-positive Klebsiella isolates among different clinical sample types.**

| **Sample Type** | **String test positive samples** |
| --- | --- |
|  | **65.43% (N=53)** |
| Tracheal Aspirate | 41.97% (34) |
| Urine | 11.11% (9) |
| Sputum | 9.88% (8) |
| Pus | 1.24% (1) |
| Wound swab | 1.24% (1) |

**References:**

1.	Sun F, Wu D, Qiu Z, Jin M, Li J. Development of real-time PCR systems based on SYBR Green for the specific detection and quantification of Klebsiella pneumoniae in infant formula. Food Control. 2010;21(4):487-91.

2.	Guerra B, Junker E, Miko A, Helmuth R, Mendoza M. Characterization and localization of drug resistance determinants in multidrug-resistant, integron-carrying Salmonella enterica serotype Typhimurium strains. Microbial Drug Resistance. 2004;10(2):83-91.

3.	Guo Y, Zhou H, Qin L, Pang Z, Qin T, Ren H, et al. Frequency, antimicrobial resistance and genetic diversity of Klebsiella pneumoniae in food samples. PloS one. 2016;11(4):e0153561.

4.	Nadasy KA, Domiati-Saad R, Tribble MA. Invasive klebsiella pneumoniae syndrome in North America. Clinical Infectious Diseases. 2007;45(3):e25-e8.
